# Growth Abnormalities of Fetuses and Infants

**DOI:** 10.1155/2017/3191308

**Published:** 2017-11-22

**Authors:** Erich Cosmi, Enrico Grisan, Vassilios Fanos, Giuseppe Rizzo, Shanthi Sivanandam, Silvia Visentin

**Affiliations:** ^1^University of Padua, Padua, Italy; ^2^University of Cagliari, Cagliari, Italy; ^3^University of Rome Tor Vergata, Rome, Italy; ^4^University of Minnesota, Minneapolis, MN, USA

The objective of this special issue is to address recent research trends and developments about the advancements of image processing and vision in healthcare. A substantial number of papers were submitted, and after a thorough peer review process, some of these were selected to be included in this special issue. Growth abnormalities (either growth restriction or large for gestational age) during perinatal and postnatal life are a hot topic issue, since they are often linked to alteration of uterine environment caused by placental insufficiency, maternal metabolic syndrome, and in general under- or overnutrition of the fetus. These fetal abnormalities account for the leading causes of perinatal morbidity and mortality. Moreover, under the hypothesis of developmental origin of adult diseases, they bear consequences in later life, programming the infant physiology for a higher risk of noncommunicable diseases, cardiovascular adult diseases, and neurodevelopment delay. Low birth weight, caused either by preterm birth and/or by intrauterine growth restriction, is recently known to be associated with increased rates of cardiovascular disease and noninsulin dependent diabetes in adult life. The “developmental origins of adult disease” hypothesis, often called “the Barker hypothesis,” proposes that these diseases originate through adaptations of the fetus when it is undernourished. These adaptations may be cardiovascular, metabolic, or endocrine and they may permanently change the structure and function of the body, increasing coronary heart disease risk factors, such as hypertension, type 2 diabetes mellitus, insulin resistance, and hyperlipidaemia. This hypothesis originally involved from observation by Barker and colleagues that the regions in England with the highest rates of infant mortality in the early 20th century also had the highest rates of mortality from coronary heart disease decades later. As the most commonly registered cause of infant death at the start of 20th century was low birth weight, these observations led to the hypothesis that low birth weight babies who survived infancy and childhood might be at increased risk of coronary heart disease in later life. There is an increased evidence of the link between intrauterine and perinatal alterations and adult diseases. Although the main focus so far has been the timing of delivery and follow-up, the study of the pathophysiology and of possible recovery is of paramount importance and needs the contributions of physicians from several fields, biologists, bioinformaticians, and engineers.

The paper by C. Caissutti and V. Berghella presents a comprehensive review about the most important and employed guidelines about screening and management of gestational diabetes (GDM) that affects up to 7% of pregnant women and is associated with several maternal and perinatal morbidities. It is well known that GDM represents over time a risk factor of type 2 diabetes, for both the mother and the fetus. Nowadays, there are many unsolved questions concerning the indications of screening, the timing and type of screening, the criteria for diagnosis, and the population to screen. The correct identification of universally approved and shared recommendations should improve the GDM pregnancy outcomes (gestational hypertension, prematurity, cesarean deliveries, number of large for gestational age and small for gestational age fetuses, and 1-minute Apgar scores < 7), improving the health care and maintaining a cost-effective benefit.

The paper by B. Chiofalo et al. takes into account the role of microRNAs (miRNAs) of the placenta in the intrauterine growth restriction (IUGR) disease. IUGR could be considered as a placentation disorder, derived from a dysregulation in trophoblast invasion with characteristic tissue morphology that leads to uteroplacental insufficiency. More than 1880 miRNAs have been reported in humans and most of them are expressed in placenta. They seem to modulate cell development, differentiation, and proliferation, cell type-specific function, and epigenetic processes. In several cases, miRNA is significantly different between physiology and pathological conditions. An abnormal upregulated placental expression regulating some angiogenic regulatory pathways seems to increase vascular resistances, also in growth-restricted human pregnancies. Despite the fact that several authors have demonstrated a relatively easy and feasible detection of some miRNAs in maternal whole peripheral blood, costs of these tests should be reduced in order to increase cohorts and have stronger evidence. A large cohort and an adequate statistical power could identify a panel of biomarkers on maternal peripheral blood for early diagnosis of IUGR.

The paper of W. M. Curtin et al. analyzes the correlation between Doppler abnormalities in fetuses with suspected IUGR delivered at 37 weeks' gestation and placental histopathological lesions, in order to remove the confounding factor that gestational age has on interpretation of placental disease. The correct definition of IUGR nowadays is object of discussion. Up to 70% of fetuses with suspected IUGR may be constitutionally small normal infants and the remainder will be classified as IUGR presumably secondary to a pathologic placental process. In IUGR at later gestational ages, abnormal umbilical artery Doppler patterns are less frequent; the placental pathology is subtler and the lesions can overlap with normal pregnancies. This aspect could distinguish the “true” or “pathologic” IUGR fetuses. In their study the authors show an association between abnormal Doppler patterns and the presence of placental pathology in singleton pregnancies delivered at 37 weeks' gestation for suspected IUGR. In particular, an abnormal MCA Doppler had the strongest association and underscores the limitation of umbilical artery Doppler alone in IUGR at later gestational ages. Further investigation and tools for separating the constitutionally small normal fetus from the IUGR fetus are needed.

The paper of M. G. Clemente et al. describes an observational retrospective study about the postnatal growth in a cohort of IUGR infants. The fetal programming theory postulates that the condition of IUGR is associated with an increased risk of cardiovascular events in adult life, as stroke, type II diabetes, metabolic syndrome, and neurocognitive impairment. At birth, significant differences were found between IUGR and controls neonates with regard to all the auxological parameters (weight, head circumference, and length). During the 1st year, 8 of 12 (70%) IUGR infants exhibited a significant catch-up growth in the 3 anthropometric parameters and a regular growth until the 3rd year of follow-up. The majority but not all infants born with IUGR in our series showed significant postnatal catch-up growth essentially during the first 12 months of life. The modern research describes a new personalized medicine approach through the Newborn Individualized Developmental Care and Assessment Program (NIDCAP), conducted on preterm infants born with severe IUGR by a multidisciplinary research working group. It seems to be effective in ameliorating the neurobehavior, electrophysiology, and brain structure outcomes. An improved knowledge of the causes of IUGR will help to develop measures for its prevention and individualized treatment.

The paper of Q. Guo et al. takes into account the congenital heart defect (CHD), one of the most common birth defects in the world. Around the world, periconceptional folic acid intake in females is thought to reduce the risk of CHD in the newborn. The methylenetetrahydrofolate reductase (MTHFR) and methionine synthase reductase (MTRR) genes are two of the most important candidate genes for fetal CHD. However, the correlations between the two genes and fetal CHD were inconsistent in various reports. This study is aimed to evaluate the parental effects of the two genes on fetal CHD via three genetic polymorphisms, MTHFR 677C>T (rs1801133), MTHFR 1298 A>C (rs1801131), and MTRR 66A>G (rs1801394). Parents with pregnancy history of fetal CHD were divided into two subgroups in base on ventricular septal defect (VSD) and non-VSD groups. In either maternal or paternal group, the MTHFR 677C>T polymorphism was independently related to fetal non-VSD, while the MTRR 66A>G polymorphism was independently related to fetal VSD. The findings of this study could help to explain why the relationship between the two polymorphisms (MTHFR 677C>T and MTRR 66A>G) and CHD varied among different studies due to different proportion of VSD and non-VSD subjects included in those different population studies.

The paper of D. Darmochwal-Kolarz et al. investigates the role of Interleukin-17 (IL-17), Interleukin-23 (IL-23), and transforming growth factor *β* (TGF *β*) in pregnancy complicated by placental insufficiency, fetal growth restriction, and preeclampsia. In recent years, in order to clarify the immunological mechanisms responsible for the proper implantation process, the interest has moved on the Th1/Th2/Th17 paradigm. Interleukin-17 (IL-17) is a cytokine with potent proinflammatory properties and has a proven role in the development of inflammatory processes. Moreover, Interleukin-23, which is produced, among others, by macrophages and dendritic cells, is an important component of the inflammatory response. Finally, transforming growth factor *β* (TGF *β*) released, among others, by macrophages, neutrophils, platelets, and lymphocytes, acts primarily to reduce the release of proinflammatory cytokines. In addition, TGF *β* is involved in the processes of angiogenesis, wound healing, and repair processes, as well as regulation of the entry of cells onto the apoptotic pathway. In the group of patients with placental insufficiency, the maternal sera levels of IL-17 positively correlated with maternal systolic blood pressure and the concentrations of TGF *β* were significantly lower, while IL-23 was comparable with respect to control group. It seems possible that the increased concentrations of IL-17 and the deficiency of TGF *β* in pregnancy complicated by fetal growth restriction and preeclampsia can be responsible for the activation of inflammatory response.

The paper of L. Baken et al. discusses the introduction of three-dimensional (3D) ultrasound in the evaluation of the crown-rump length (CRL), in particular whether the embryonic volume (EV), measured by a Virtual Reality (VR) system, is a better parameter to determine growth restriction in fetuses with structural congenital abnormalities, diagnosed in the first trimester of pregnancy. It is know that the relative increment of the EV is much larger than the increment of the CRL during the same period. Moreover, if a too small CRL is a clinical predictor for miscarriage, chromosomal abnormalities (especially trisomy 18), and fetal growth restriction in the second and third trimester of pregnancy, several authors underlined that EV was not only smaller in trisomy 18 pregnancies but also in trisomy 21 and trisomy 13 pregnancies. In this study, measured CRL and EV were converted to *z*-scores and to percentages of the expected mean, using published reference curves of euploid fetuses. The EV was smaller than expected for gestational age in fetuses with structural congenital abnormalities, whereas CRL was not. By measuring EV, first-trimester growth restriction becomes more evident and might enable an earlier detection of cases at risk for a congenital abnormality.

The paper of F. Bardanzellu et al. presents a comprehensive review of paracetamol efficacy in Ductus Arteriosus (DA) closure in preterm infants, in which the failure or delay of its spontaneous closure results in the condition of Patent Ductus Arteriosus (PDA). A prolonged situation of PDA can be associated with several short- and long-term complications. Despite years of researches and clinical experience on PDA management, unresolved questions about the treatment and heterogeneity of clinical practices still remain, in particular regarding timing and modality of intervention. Nowadays, the most reasonable strategy seems to be reserving the treatment only to hemodynamically significant PDA. The first-line therapy is medical, and ibuprofen, related to several side effects especially in terms of nephrotoxicity, is the drug of choice. Administration of oral or intravenous paracetamol (acetaminophen) recently gained attention, appearing effective as traditional nonsteroidal anti-inflammatory drugs (NSAIDs) in PDA closure, with lower toxicity. The results of the studies analyzed in this review mostly support paracetamol efficacy in ductal closure, with inconstant low and transient elevation of liver enzymes as reported side effect. More studies are needed to confirm if this therapy shows a real safety profile and to evaluate its long-term outcomes.

The paper of R. Pintus et al. discusses the most recent literature about the metabolomic technology and its application in the cardiologic field, in order to understand the metabolic shifts that occur even before the manifestation of heart diseases and to find possible early predictive biomarkers. Cardiac pathologies are a critical health issue affecting millions of people worldwide, with a constant mortality rate in particular in the elderly, a difficult prognosis, and a worsening in quality of life of affected people. The pathophysiology of heart pathologies is complex. Recent findings pointed out a possible pivotal role of mitochondrial dysfunction and the subsequent altered energy metabolism in cardiac diseases, in particular in case of heart failure. Moreover, every adverse event that may occur during pregnancy “shapes” the health status of the fetus and its development and could affect its life course, also with cardiovascular problems. Congenital malformations, gut colonization by microbiota, individual genetic arrangement, and its interplay with both behavioral and risk factors, such as drugs assumption, can influence the occurrence of heart diseases. During the last decade, animal and human studies have applied metabolomics to cardiovascular research, using both targeted and untargeted approaches; as such, metabolic fingerprints have been identified for several cardiovascular risk factors and diseases. These techniques could be applied to heart tissue and biofluids, such as blood, saliva, and urine, with a minimum compliance needed from the patient, since their collection is not invasive. Metabolomics, for its peculiarities, seems to be so promising that several industries are trying to set up kits to immediately assess the metabolites variations in order to provide a faster diagnosis and the best treatment specific for that patient, offering a further step toward the path of the development of a tailored medicine.

The paper of N. Abdalla et al. describes complete up-to-date findings from the literature regarding the impact of chemotherapy on fetal growth. Cancer and pregnancy rarely coincide. Gynecological cancers are among the most common malignancies to occur during pregnancy, and chemotherapy with or without surgery is the primary treatment option. The main concern of administering chemotherapy during pregnancy is congenital malformation, although it can be avoided by delaying treatment until after organogenesis. The dose, frequency, choice of chemotherapeutic agents, time of treatment commencement, and method of administration can be adjusted to obtain the best maternal treatment outcomes while simultaneously minimizing fetal toxicity. Use of chemotherapy after the first trimester, while seemingly safe, can cause fetal growth restriction. However, the exact effect of chemotherapy on such fetal growth restriction has not been fully established; information is scarce owing to the rarity of malignancy occurring during pregnancy, the lack of uniform treatment protocols, different terminologies for defining certain fetal growth abnormalities, the influence of mothers' preferred options, and ethical issues. Fetal growth abnormalities are recognized sequelae of chemotherapy, and the possibility of fetal growth abnormalities as well as the other side effects of different chemotherapeutic agents should be discussed with the patients.

The type of malignancy and its stage, the use of surgery and/or radiotherapy, the stage of pregnancy, the probability of side effects during treatment, and the patient's own wishes influence the final decision regarding treatment. Moreover, ethical concerns cannot be ignored. Each patient should ultimately be managed individually with the guidance of a multidisciplinary team.

The paper of S. A. Ernst et al. investigates what care-related and maternal risk factors could influence the antenatal nondetection of IUGR during pregnancy and examine if there are specific groups with a higher chance of nondetected suboptimal fetal growth. Approximately 3% to 8% of all infants born in developed countries have been identified as growth restricted. IUGR is a prenatal condition associated with a higher risk for perinatal morbidity and mortality, increasing the stillbirth rate fourfold compared to pregnancies with normally grown fetuses; antenatal nondetection further increases the rate by a factor of 2. An early antenatal detection, choosing the optimal time and method of delivery, and treatment where appropriate could minimize the risks significantly. However, low antenatal detection rates of suboptimal fetal growth through routine fetal ultrasonography have been reported. In fact IUGR has been reported to be antenatally detected only in one-third (25% to 32%) of pregnancies with suboptimal fetal growth. Moreover, the authors identified three factors that influenced IUGR detection: a higher severity of the growth restriction, maternal complications/diseases during pregnancy, and a Doppler examination. The authors did not find statistically significant differences regarding parental socioeconomic status and maternal migration background. Future in-depth studies with larger study populations should further examine factors that could increase antenatal detection rates for IUGR.

The paper of S. Norda et al. discusses the role of neonatal ApoE e4 haplotype on IUGR and its contribution on impaired fetal growth and the possible link of IUGR with cardiovascular and metabolic diseases later in life. Apolipoprotein E (ApoE) is an important circulating serum protein involved in transporting lipids and cholesterol and regulating lipid levels. Its regulatory functions have been attributed to many biophysiological processes including neuronal growth and modulation of oxidant and inflammatory processes. Cord blood of IUGR neonates displays lipid changes towards atherosclerotic profiles. IUGR born babies were found to have lower concentrations of high-density lipoprotein cholesterol (HDL-C), known for having an anti-inflammatory effect and protective properties against the development of atherosclerosis, while triglycerides and oxidized low-density lipoprotein (oxLDL) levels were elevated in samples of umbilical blood compared to adequate weight newborns. This disrupted cholesterol and triglyceride handling plays a role in causing suboptimal fetal development and exposes the newborn to an atherosclerotic environment early, consequentially giving raise to irreversible damage to vessels. The association of ApoE e4 with the development of dyslipidemia and cardiovascular disease is known. In newborns, carrying the ApoE e2 allele has been associated with lower fetal cord blood LDL-C levels and higher levels of HDL-C suggesting a beneficial effect of this genotype on blood lipid configuration, modulating the fetal growth and the severity of IUGR. In this cohort, 4885 preterm infants were analyzed. Neonates were categorized into subgroups of <3rd, 3rd–10th, and >10th birth weight percentile. The identification of the ApoE genotype was carried out. No association was found between genotype and birth weight percentiles in each of the subgroups. ApoE genotype and low birth weight depict two distinct risk factors for cardiovascular disease without being directly associated.

The paper of M. Gatta et al. describes that prematurity has a critical influence on interactive, communicative, and expressive child behaviour, particularly during the first years of life. Few studies have stressed the assessment of mother-father-child interaction in families with preterm children, generating contradictory results. The authors recruited 78 families, 39 families with preterm children, and 39 families with full-term children. Results show that families with preterm children display a low quality of mother-father-child interactions. After six months, family interactions result is generally stable, except for some Lausanne Trilogue Play-scales, reflecting a hard adjustment of parenting style to the evolution of the child. The Lausanne Trilogue Play is a semistandardized observation situation designed to assess the quality of family interactions. The administration involved the mother-father-child triad invited to cooperate and work together in order to conduct an activity. In families with preterm children, the parenting stress seemed to be correlated with the quality of mother-father-child interactions.

